# Trichobezoar: A Rare Cause of Protein-losing Enteropathy

**DOI:** 10.1097/PG9.0000000000000310

**Published:** 2023-04-18

**Authors:** Anam Bashir, Andrea Gosalvez Tejada, Keith T. Oldham, Pooja D. Thakrar, Diana G. Lerner

**Affiliations:** From the * Division of Pediatric Gastroenterology, Hepatology, and Nutrition, Department of Pediatrics, Medical College of Wisconsin, Milwaukee, WI; † Division of Pediatric Surgery, Department of Surgery, Medical College of Wisconsin, Milwaukee, WI; ‡ Division of Pediatric Radiology, Department of Radiology, Medical College of Wisconsin, Milwaukee, WI.

## Abstract

Protein-losing enteropathy (PLE) is caused by protein loss through the gastrointestinal tract which results in hypoalbuminemia. The most common causes of PLE in children include cow milk protein allergy, celiac disease, inflammatory bowel disease, hypertrophic gastritis, intestinal lymphangiectasia, and right-sided heart dysfunction. We present a case of a 12-year-old male with bilateral lower extremity edema, hypoalbuminemia, elevated stool alpha-1-antitrypsin, and microcytic anemia. He was found to have a trichobezoar in the stomach extending to the jejunum, an unusual cause of PLE. The patient underwent an open laparotomy and gastrostomy to remove the bezoar. Follow-up confirmed resolution of hypoalbuminemia.

## INTRODUCTION

Bezoar is a mass composed of indigestible material that develops within the gastrointestinal tract. The most common site of development is the stomach. Bezoars can be compromised of vegetable matter (phytobezoar), fruit fibers, hair (trichobezoar), milk (lactobezoar), stones, plastic, yarn, tissue paper, cement, vinyl gloves, or pills (pharmacobezoar). Bezoars can be asymptomatic or can present with abdominal pain, nausea, vomiting, anorexia, gastrointestinal bleeding, weight loss, abdominal distension, or mass (1). Severe protein-losing enteropathy (PLE) with hypoalbuminemia, edema, and anemia is a rare presentation of trichobezoar.

## CASE

**FIGURE 1.**
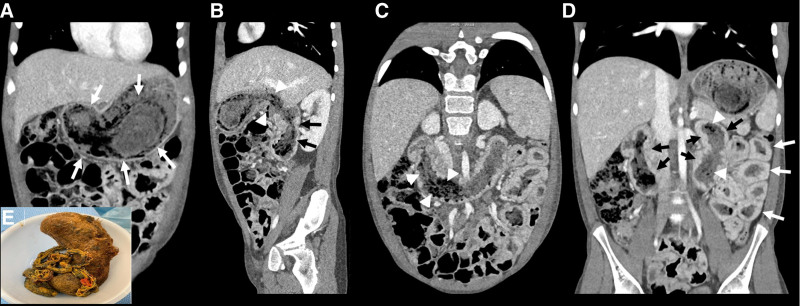
Coronal (A) and sagittal oblique (B) contrast-enhanced computerized tomography (CT) images of the abdomen and pelvis revealed a large, mixed attenuation mass distending the gastric lumen (white arrows) and extending through the pylorus (white arrowheads) into the proximal duodenum (black arrows). Coronal (C) and sagittal oblique (D) contrast-enhanced CT images of the abdomen and pelvis revealed a large, mixed attenuation mass distending the gastric lumen (white arrows) and extending through the pylorus (white arrowheads) into the proximal duodenum (black arrows). (E) Surgically extracted trichobezoar forming a cast of stomach, duodenum, and jejunum.

A 12-year-old previously healthy male who weighed 32.7 kg (7.92 percentile, Z score −1.41) presented to the pediatrician’s office with a 3-month history of bloating and fatigue and a 2-week history of the bilateral lower extremity and scrotal edema. Physical examination was significant for abdominal distention and bilateral lower extremity edema to the knees. He had normal vital signs. Laboratory evaluation revealed hemoglobin of 11.5 g/dL (12.1–16.1 g/dL), hematocrit of 34.9% (36–47%), mean corpuscular volume of 87.5 fL (78–95 fL), and albumin of 1.5 g/dl (3.8–5.4 g/dL). Urinalysis was negative for protein. Serum iron level was 40 ug/dL (44–142 µg/dL), total iron binding capacity was 155 mg/dL (224–435 mg/dL), and ferritin was 9 ng/mL (10–300 ng/mL), consistent with iron deficiency. Other laboratory evaluations showed normal liver and kidney function. The patient was transferred to our emergency room, where he received a 0.5 g/kg infusion of 25% albumin followed by a dose of furosemide. Abdominal ultrasound showed a small bowel to small bowel intussusception within the left upper abdominal quadrant without signs of ischemia. Computed tomography (CT) of the abdomen revealed a mass filling and distending the stomach and extending through the proximal small bowel to the level of the proximal jejunum. Diffuse wall thickening and mucosal hyperenhancement were noted along the duodenum and jejunum (Fig. 1). Small ascites and diffuse subcutaneous edema were also present. The patient underwent exploratory laparotomy for delivery of the bezoar through a gastrotomy (Fig. 1E). The gastric portion of the trichobezoar measured 16 × 5 × 5 cm and retained the shape of the stomach. The small bowel extension of the bezoar measured 141 cm in length. Stool alpha-1-antitrypsin collected on admission was elevated at 51.5 mg/g (<2.1 mg/g), confirming PLE. Psychology was consulted during the admission. The patient was discharged home on ferrous sulfate. He was seen in the clinic for follow-up and remains asymptomatic without recurrence of edema.

## DISCUSSION

Excessive protein loss through the gut results in PLE. The primary symptom of PLE is edema caused by hypoalbuminemia. Erosive mucosal disease, anomalies of the lymphatic system, and right-sided cardiac failure are the most common causes of PLE. Other etiologies, such as decreased protein synthesis or greater loss through the skin, liver, or kidneys, should be considered in patients with hypoalbuminemia. Alpha-1-antitrypsin concentration in the stool is used to confirm the diagnosis of PLE (2,3). PLE has been previously described in the literature as a rare presentation of trichobezoar (4,5). In our patient, we speculate that the tail of the bezoar extending into the small bowel caused mucosal irritation in the duodenum and small bowel. In conjunction with probable early satiety and decreased food intake resulting from the space-occupying bezoar within the stomach, this led to hypoalbuminemia and low iron levels. It is also possible the large trichobezoar caused lymphatic obstruction which could have led to increased protein leakage (3).

Trichobezoar is a mass of hair, fur, or fiber which accumulates within the gastrointestinal tract. A gastric trichobezoar that extends through the pylorus is also sometimes referred to as “Rapunzel syndrome” (1). Bezoars are typically seen in adolescents with a female predominance. Patients can have an underlying psychiatric disorder (5,6). The tail of the mass can extend into and through the small intestine as far as the cecum and may cause enteritis. The presentation varies according to the bezoar’s duration, volume, and location. The most typical signs and symptoms include nausea, vomiting, weight loss, anorexia, abdominal pain, discomfort or fullness, abdominal mass, bloating, and difficulty swallowing (1,5–8). Patients may develop mucosal ulcers or necrosis, which can result in anemia, bloody stools, and melena or hematemesis (1,9). In rare cases, these can also lead to gastric or intestinal perforation, gastric outlet obstruction, or intussusception (10,11). Hypoalbuminemia and edema associated with trichobezoar are rare (3–6,8,10–13) (Table 1).

**TABLE 1.** Cases of trichobezoar presenting with edema and hypoalbuminemia

Case	Publication	Age/Sex	Patient presentation	Finding	Removal
1.	Rabie et al. (2008)	11 year/F	Epigastric pain, abdominal mass, vomiting, and hypoalbunemia	Trichobezoar extending to the jejunum	Surgical
2.	Vila et al. (2009)	16 years/F	Epigastric pain, vomiting, abdominal mass, anemia, and severe hypoalbunemia (1.7 g/dl)	Trichobezoar extending to duodenum	Surgical
3.	Prasanna et al. (2013)	16 years/F	Abdominal pain, vomiting abdominal mass, anemia, hypoalbunemia	Trichobezoar extending to the ileum	Surgical
4.	Nour et al. (2017)	4 year/F	Edema of lower limbs, epigastric mass, microcytic hypochromic anemia	Trichobezoar extending to the jejunum	Surgical
5.	Malhotra-Gupta et al. (2017)	10 year/F	Abdominal pain, decreased appetite, weight loss, anemia, hypoalbuminemia (1.9 g/dl)	Trichobezoar in stomach	Surgical
6.	Stinco et al (2020)	7 years/F	A 2-week history of anasarca, microcytic hypochromic anemia with severe hypoalbuminemia (1.8 g/dl)	Trichobezoar extending to jejunum	Surgical
7.	Kaufman et al. (2020)	4 years/F	Periorbital and lower extremity swelling, vomiting, diarrhea, iron deficiency anemia, hypoalbunemia(1 g/dl)	Trichobezoar extending to the duodenum	Surgical
8.	Riveros-Vega et al. (2020)	35 year/F	Early satiety, loss of weight, hypoalbunemia, microcytic anemia,	Trichobezoar extending to the jejunum	Surgical
9.	Keyur et al. (2021)	15 years/F	Abdominal pain, bilateral pedal edema, abdominal mass,	Trichobezoar extending to the ileum	Surgical

CT is the imaging of choice for diagnosing bezoars and may show a circumscribed mass with varying densities and air pockets (6). Endoscopy may be used to make a conclusive diagnosis and can help determine the consistency and size of the bezoar. Treatment involves the removal of the mass. Endoscopic removal can be attempted if the bezoar is small; however, it is estimated that only 5% of attempted endoscopic removals are successful (9).

Underlying anatomic abnormalities such as pyloric stenosis, gastroparesis, medications that decrease motility, and excessive fiber consumption increase the risk of bezoars. Risk factors for the development of trichobezoar particularly include trichotillomania and the coexistence of psychiatric disorders (1).

Different techniques are described in the literature to remove the bezoar like fragmentation of the bezoar with different endoscopic devices such as biopsy forceps, polypectomy snare, and endoscopic scissors, and then retrieval of fragmented pieces. Retrieval may need multiple endoscopic passes and a prolonged procedure time of up to 5 hrs. We have summarized case reports (Table 2) that have described the successful removal of trichobezoar endoscopically (14–23). These case reports are, however, outnumbered by cases reported where endoscopic removal was unsuccessful. Endoscopic removal could be considered in patients where parents refuse surgical treatment or surgery is contraindicated. However, endoscopic removal is often prolonged and requires an expert endoscopist to complete it safely. Multiple endoscopic passes pose the risk of pharyngeal and esophageal perforation and prolonged sedation. Also, success rates increase if the trichobezoar is small and is totally intragastric. Trichobezoars often have synthetic fibers mixed where electrical endoscopic devices if used can release toxic fumes. Management decisions should be made case by case. Surgeons should be on standby if the decision is made to attempt endoscopic removal in case of unforeseen complications or failure to remove after endoscopy.

**TABLE 2.** Cases of trichobezoar removed endoscopically

Series No.	Authors	Age/Sex	Extension of trichobezoar	Method	Duration of procedure	Complication
1.	Soehendra N et al. (1989)	17 y/F	Gastric trichobezoar (15 cm × 7 cm)	Initial scope cutting with Nd: neodymium-doped yttrium aluminum garnet lengthwise, small fragments were removed Dormia basket	3 sessions (2–3 hrs. each)	
2.	Aybar et al. (2009)	5 y/F	Trichobezoar in the stomach extending through duodenal bulb (8 cm × 7 cm)	Bezoar was broken into 13 smaller pieces with hot biopsy forceps and snare, required 25 passes	3 hrs	Mucosal abrasions, small superficial mucosal burns in the greater curvature
3.	Konuma et al. (2011)	9 y/F	Trichobezoar extending to the duodenum (1.8 cm × 3.2 cm × 34 cm)	Grasper and net used to retrieve the trichobezoar	15 min	None
4.	Kao et al. (2015)	5 y/F	Gastric trichobezoar	Enmass removal	Extubation during the procedure	
5.	Benatta et al. (2016)	6 y/F	Gastric trichobezoar with few extensions through the pylorus (8 cm × 4 cm)	Bezoar was fragmented into pieces using polypectomy snare and argon plasma coagulation. Total of 15 passes after breaking the trichobezoar into 10 pieces	50 min	Fundic erosions, mucosal polypoid ulcerations of greater curvature
6.	Zhao et al. (2017)	12y/F	Gastric trichophytobezoar with an extension of a few hairs through the pylorus (10.5cm x 3.5 cm)	Hard core made of mixed fibers and vegetable material was cut with endoscopic scissors, trichophytobezoar was fragmented into pieces with polypectomy snare and APC, hair and nondigestible food fibers were removed with grasping forceps, remaining bezoar was loosened with biopsy forceps and was injected with 50 mL of 5% sodium bicarbonate solution through the biopsy channel. Second endoscopy was done after 5 days. Hairball was cut with scissors and then fragmented with a polypectomy snare and APC	Two endoscopy sessions 5 days apart	None
7.	Allam et al. (2019)	4 y/F	Gastric trichobezoar (16cm × 10 cm)	Fragmentation of the bezoar by foreign body forceps with the removal of a small piece by piece, 100 endoscopic passes	5.5 hrs	None
8.	Wang et al. (2021)	9 y/F	Gastric trichobezoar	An overtube channel was created with a plastic sheath between the oral cavity and stomach to facilitate the reintroduction. Forceps and snare were used to fragment the trichobezoar.		
9.	Wang et al. (2021)	14 y/F	Gastric trichobezoar (12cm × 6cm)	Polypectomy snare was used to break the trichobezoar, hook knife was used to fragment the trichobezoar, removed by dormier- type stone retrieval basket, removed in three endoscopy passes	1 hr.	None
10.	Lu D et al. (2022)	11 y/F	Gastric trichobezoar and duodenal strands of hair (10 cm × 10 cm)	Variceal ligator cap was used to position strands of hair and grasping forceps was used to loosen the hair. Grasping forceps and polypectomy snare were used to grasp hair strands into the variceal ligator cap. Hair strands were removed in small pieces. Required 20 passes	5.5hrs	Mucosal abrasions in the esophagus

## ACKNOWLEDGMENT

Informed consent was obtained from the patient’s parent before the submission of this case for publication.
